# The Relationship between Microbial Community Succession and Flavor Formation during the Natural Fermentation of Hongqu sufu

**DOI:** 10.3390/foods12142800

**Published:** 2023-07-23

**Authors:** Aiguo Luo, Zilong Cheng, Jia Zhao, Jianwei Hao, Shengli Shi, Bianfang Hu

**Affiliations:** 1 Department of Biological Science and Technology, Jinzhong University, Jinzhong 030619, China; lag@jzxy.edu.cn (A.L.); zhaojia@jzxy.edu.cn (J.Z.); haojianwei@jzxy.edu.cn (J.H.); shishengli@jzxy.edu.cn (S.S.); 2 College of Life Sciences, Hubei University, Wuhan 430062, China; 13277424479@163.com

**Keywords:** Hongqu sufu, microbial communities, flavors, correlation analysis

## Abstract

To study the diversity of microbial flora in Hongqu sufu and analyze the characteristics of special flavor compounds, this study took self-made Hongqu sufu as the research object. Dynamic changes in sufu during fermentation were studied. High-throughput sequencing (HTS) was used to analyze changes in the diversity of fungal and bacterial communities during fermentation. The results showed that at the phylum level, the dominant fungal phyla were identified, Mucormyces and Ascomycetes. The dominant bacterial phyla were Proteobacteria and Firmicutes. At the genus level, the dominant fungal genera were identified as *Actinomucor*, *Monascus,* and *Aspergillus*. The dominant bacterial genera were *Pseudomonas*, *Aneurimibacillus*, *Sphingobacterium*, and *Bacillus*. Headspace solid-phase microextraction gas chromatography–mass spectrometry (HS-SPME-GC-MS) combined with technology that can dynamically change flavor compounds was explored to investigate the correlation between microbiota and flavor compounds. In different stages of fermentation, 75 main volatile organic compounds were identified, including seven alcohols, four acids, 16 alkanes, 14 olefins, seven kinds of aldehydes, two kinds of ketones, 10 kinds of esters, one kind of phenol, one kind of sulfur-containing compound, one benzene, and 12 other compounds. The correlation analysis between flora and flavor compounds showed that the fungi genera *Alternaria* and *Pichia* were significantly correlated with most flavor compounds. Bacteria genera including *Weissella*, *Hafnia-Obesumbacterium*, and *Leuconostoc* had a strong positive correlation with ethyl oleate.

## 1. Introduction

Sufu is an original condiment in China and is a traditional folk food with a unique flavor that has a history of thousands of years. It has good taste, rich nutrition, and unique taste, which has led to it gaining popularity among the Chinese people and people in Southeast Asian countries [1,2]. In terms of China’s sufu consumption, Hongqu sufu has the highest proportion, accounting for 48% of sufu consumption in 2022. It is a famous sufu brand with annual sales reaching 25210 tons. The fermentation process of sufu is a process involving the succession of microbial communities, during which these microorganisms produce various enzymes that make sufu easier to absorb in the body [3]. In initial studies of the structural diversity of microbial flora, traditional methods were mainly used as research methods; however, when studying more complex microbial flora, traditional methods could not fully detect microbial flora, and it was easy to underestimate the number and diversity of microflora [4,5,6]. With the rapid development of microbiota microbial sequencing technology, the current effective method for detecting sufu microbiota is high-throughput sequencing (HTS), which is currently a powerful tool for assessing the diversity of microbial flora [7,8]. Xu Qiong et al. [9] used HTS to sequence the genes in the 16 SrDNAV1-V3 regions of the Hongqu sufu strain to analyze the bacterial diversity in Hongqu sufu in different regions, and the research deepened the understanding of bacterial community composition and diversity in Hongqu sufu. Many studies have applied this technique to the analysis of microbial diversity during fermentation, such as in tempeh [10], bean juice [11], miso [12], Qula [13], and so on. In terms of research on sufu flavor compounds, Ran Chunxia et al. reported the following finding [14]: the extraction and analysis of volatile compounds of sufu by headspace solid-phase microextraction gas chromatography–mass spectrometry technology combined with headspace solid-phase microextraction gas chromatography–mass spectrometry (HS-SPME-GC-MS) technology indicates that HS-SPME-GC-MS technology is most mature for the analysis of flavor compounds in sufu. At present, there have been relatively few studies on the structure and flavor characteristics of Hongqu sufu, so it is necessary to conduct in-depth research on the diversity of microorganisms in sufu and the correlation between microbiota structure and its flavor.

In this study, the microbial flora information in Hongqu sufu was classified and compared using HTS, the flavor compounds were extracted using the solid-phase microextraction method, and HS-SPME-GC-MS was used in parallel. Flavor compounds analysis was carried out to explore the relationship between microbial flora and flavor compounds, which was conducive to an in-depth understanding of the composition of microorganisms, bacteria, fungi, and flavor compounds in Hongqu sufu. This study lays the foundation for elucidating microbial community structure and flavor compounds analysis in sufu and at the same time contributes toward determining the dominant flora in Hongqu sufu, which provides a theoretical basis for fermented foods comprising Hongqu sufu.

## 2. Materials and Methods

### 2.1. Methods

#### 2.1.1. Experimental Treatment

Materials preparation: 6 bamboo grates, 9 pieces of gauze, 600 g of tofu (purchased from Shanxi Jinzhong Kangjin Food Company Limited, Jinzhong, China), 5 g of mucor koji power (purchased from Yishui Jinrun Biotechnology Company Limited, Linyi, China), 5 g of red yeast powder (Shanghai Jiajie Natural Food Coloring Company Limited, Shanghai, China), 100 g of chili noodles, 5 g of Sichuan peppercorn noodles, 80 g of salt, and 40 mL of high liquor were obtained. Culture preparation: All tofu supplies that were handled were boiled to ensure they were sanitized as well as oil- and water-free. Steps for the fermentation of sufu: mix mucor koji powder with sterile water at a ratio of 1:20 to form a bacterial suspension, then take 600 g of tofu and cut the tofu into blocks 3 cm × 3 cm in width and length and 3 cm in height. Soak the tofu blocks in the bacterial solution for 0.5 min to fully contaminate them with the bacterial solution before then placing the tofu blocks in a bamboo grate covered with gauze in the dark with normal room temperature maintained at about 20–25 °C. After 48 h, the tofu grows uniform white hyphae, soak the tofu cubes in 40 mL of white wine for 1 min and add the mixture of chili noodles, peppercorn noodles, salt, white wine, red yeast power, and so on. Put the cubes it in a sealed glass bottle that has been boiled to sanitize it and put the tofu on a bamboo grate covered with gauze. Place the cubes away from light and take samples every 3–6 days.

Sampling: Sampling was performed on the 1st, 3rd, 6th, 12th, and 18th days of fermentation, taking 2.0 g of experimental samples each time, and samples were then stored in centrifuge tubes. Each set of experiments was repeated three times and the samples in each group were parallelized three times, for a total of three experimental samples.

#### 2.1.2. High-Throughput Sequencing

##### Nucleic Acid Extraction, Sample DNA Extraction, and PCR Amplification of Sufu

Nucleic acid extraction: The genomic DNA of Hongqu sufu samples was extracted using the sodium lauryl sulfate method, and the purity and concentration of the sample DNA was then detected through agarose gel electrophoresis (BG-gdsAUTO gel imaging system). An appropriate amount of the sample was used. The DNA was placed in centrifuge tubes and diluted to 1 ng/μL using sterile water.

Sample DNA: an appropriate amount of sample DNA was extracted in a centrifuge tube, the experimental sample was diluted with sterile water to 1 ng/μL, and at least 3 parallel tests were performed among the groups in this experiment.

PCR amplification: Using the diluted sample genomic DNA as a template, the standard bacterial 16SV3V4 region selected the pre-primer sequence ACTCCTACGGGAGGCAGCCA and the post-primer sequence ACTCCTACGGGAGGCAGCA, which amplified V3 and V4 regions in the DNA. The standard fungal ITS1 region selected the pre-primer sequence GGAAGTAAAAGTCGTAACAAGG and the post-primer sequence GGAAGTAAAAGTCGTAACAAGG. We used specific primers with barcodes (Shanxi Saiqunsi Biotechnology Co., Ltd., Xi’an, China); Q5 High-Fidelity DNA Polymerase PCR Master Mix with GC Buffer [15]; and efficient high-fidelity enzymes for PCR to ensure amplification efficiency and accuracy [16].

#### 2.1.3. Volatile Flavor Compounds Analysis

We weighed 10.0 g of the Hongqu sufu sample, sealed it in a 20 mL headspace vial, and equilibrated it at 80 ° at a stirring rate of 500 r/min for equilibration. We stirred and heated for 35 min. We allowed headspace adsorption for 40 min, with desorption at 250 °C. An HS-SPME-GC-MS injector was inserted after 5 min [17]. The HS-SPME-GC-MS chromatographic conditions were as follows: Column: HP-5MS (30 m × 0.25 mm, 0.25 μm). Starting temperature: 50 °C, which was held for 2 min; then 5 °C, at a rate of 180 °C/min, which was held for 5 min; and then a rise to 10 °C at a rate of 250 °C/min, which was held for 5 min. Inlet temperature: 250 °C. The carrier gas was helium (He) and the carrier gas flow rate was 1.2 mL/min. There was no diversion. Constant pressure was 12 psi. Constant flow was 1.2 mL/min. Transmission line temperature was 280 °C. Ion source temperature was 230 °C. Quadruple rod temperature was 150 °C. The ionization mode was EI. Electron energy was 70 eV. Quality scanning range was 33–500 [18].

### 2.2. Statistical Analyses

All trials were conducted three times. We used IBM SPSS Statistics 26 to perform statistical analyses. We performed analysis of variance on the obtained data, which identified that *p* < 0.05 indicated significant differences. HTS results were obtained using Classifiable Operating Unit (OTU) clustering [19] to study the diversity of microbial flora. HS-SPME-GC-MS technology detected flavor compounds and we searched through mass spectrometry libraries to determine the main components of Hongqu sufu flavor compounds. Data plots were drawn using Origin 2022 and tables were drawn using Excel.

## 3. Results and Analysis

### 3.1. Microbial Species’ Diversity Curves in Sufu

**Figure 1:**
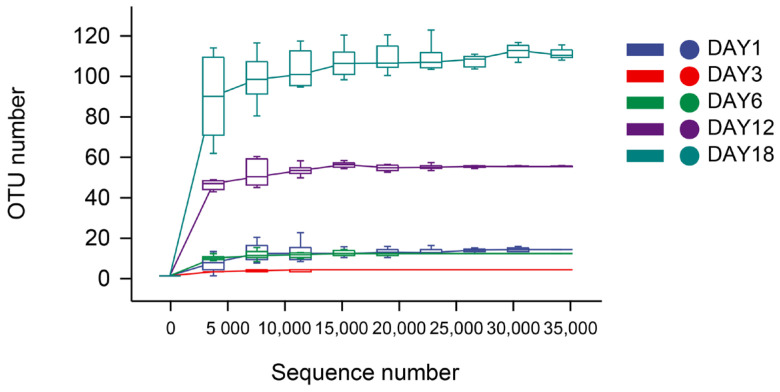
Fungal dilution curve.

**Figure 2:**
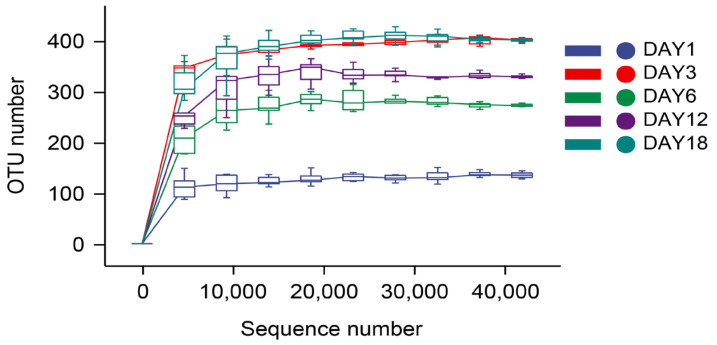
Bacterial dilution curve.

**Figure 3:**
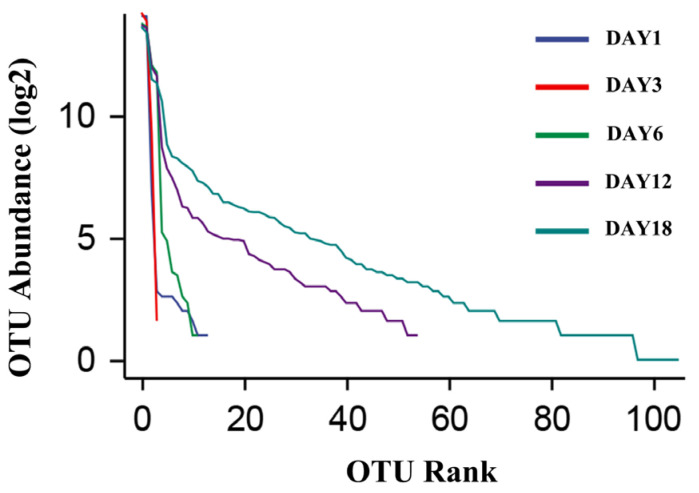
Fungal rank–abundance curve.

**Figure 4:**
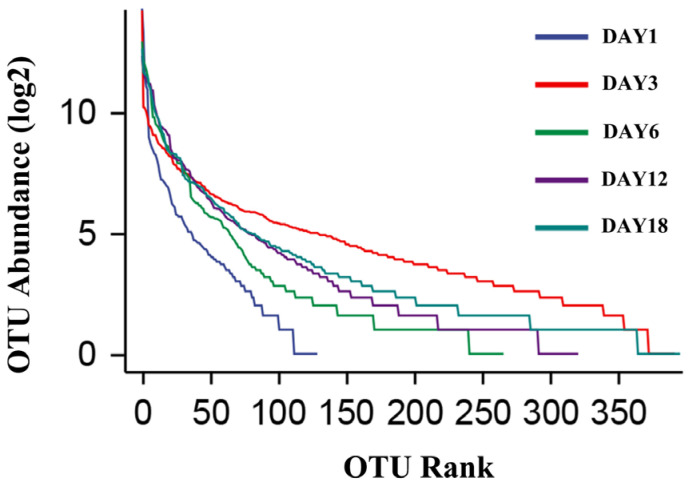
Bacterial rank–abundance curve.

The dilution curve and the rank–abundance curve was obtained in the HTS detection results as the sequencing volume of each sample of sufu was different in terms of the microbial diversity of the different sequencing [20]. The dilution curve was constructed by using the diversity index of sufu microbiota; the flatter the dilution curve, the more sufficient the sample size was. The fungal (Figure 1) and bacterial (Figure 2) dilution curves indicated that the experimental data for Hongqu sufu in the detection could fully reflect the diversity of microbial flora. The rank–abundance curve can reflect the relative abundance and distribution of microbial flora in Hongqu sufu during various stages of fermentation. The shape of curve B reflects the uniformity of the composition of microbial species. If the shapes of the two curves were similar, this meant that the distribution degree of species composition in sufu during fermentation was similar. The length of the horizontal axes of the curves is a reflection of species richness on fermentation day 18, as can be seen from Figure 3 and Figure 4. The horizontal axes of the sufu curves in Figure 3 and Figure 4—the ratio of fermentation—were wider than the curves on the twelfth, sixth, third, and first days of fermentation. Moreover, the curve for the twelfth day of fermentation was better than the curves from days one, three, and six (Figure 3 and Figure 4), as shown through the width of the horizontal axes. The results showed that the microbial composition of sufu on the eighteenth day of fermentation was higher than that on the twelfth, sixth, third, and first days of fermentation, showing that the sufu was richer. Moreover, the sufu on the twelfth day of fermentation was richer than it was on fermentation days one, three, and six. The richness of its microbial composition will become more abundant as the fermentation time increases. Obvious differences were observed in sufu’s microbial diversity at the different fermentation times.

### 3.2. Distribution of OTUs in Sufu Species and Microbial Species

**Figure 5:**
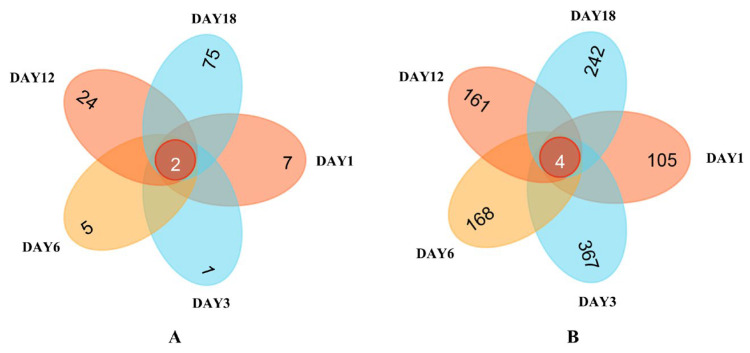
Venn diagram of the OTU distribution of fungi (**A**) and bacteria (**B**) in fermented sufu samples.

**Figure 6:**
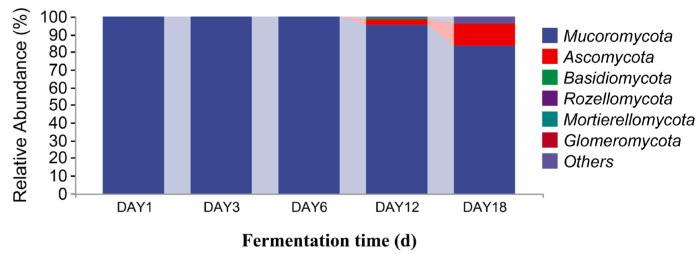
Distribution of fungal community structures in fermented Hongqu sufu samples at the phylum level.

Venn plots were obtained through HTS detection (see the Venn diagrams in Figure 5). The figures visually show the unique and common OTU numbers in the Hongqu sufu samples, and they express similarities and overlaps in the OTU number compositions of the samples [21]. As can be seen from Figure 5, on fermentation days 1, 3, 6, 12, and 18, there were two overlapping fungal OTUs in the Hongqu sufu samples, accounting for approximately 1.8% of the total OTUs. The fermentation of the sufu fungus samples on the first, third, sixth, twelfth, and eighteenth days of fermentation was unique. The number of OTUs on these days were 7, 1, 5, 24, and 75, respectively. Minor differences in fungal microorganisms among the three samples were illustrative. The number of overlaps in the bacterial OTUs, shown in Figure 6 was four, which accounted for approximately 0.38% of the total OTUs. On the first, third, sixth, twelfth, and eighteenth days of fermentation, the numbers of OTUs in the sufu bacteria were unique, at 105, 367, 168, 161, and 242, respectively. This indicated that, on the days 1, 3, 6, 12, and 18 of fermentation, there were certain differences in the bacterial microorganisms. The flavor difference between the sufu on the third day of fermentation and the sufu on the twelfth day of fermentation was obvious, which implies that the flavor compounds in the sufu are mainly determined by unique microbial flora.

### 3.3. Analysis of Microbial Species Differences in Sufu

**Figure 7:**
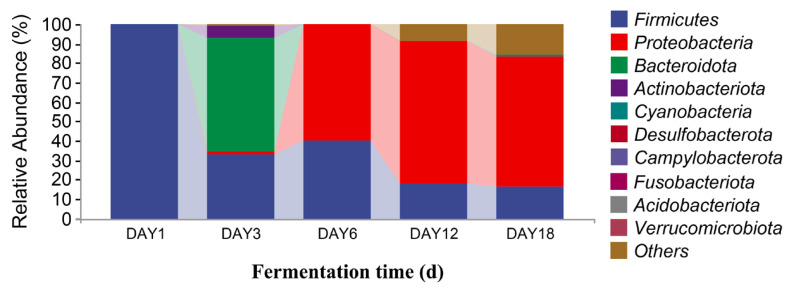
Distribution of bacterial community structures in fermented Hongqu sufu samples at the phylum level.

The sequencing data were classified at the phylum and genus levels to study the microbial community succession of Hongqu sufu at different stages of fermentation. As can be seen from Figure 6, at the phylum level, there were six fungal phyla in the fermentation process of Hongqu sufu: Mucoromycota, Ascomycota, Basidiomycota, Rozellomycota, Mortierellomycota, and Glomeromycota. The average relative abundance of Mucoromycota and Ascomycota on the first day of fermentation was 99.88% and 0.11%, respectively. The average relative abundance of Mucoromycota on the third day of fermentation was 99.99%. The relative abundance of Mucoromycota on the sixth day of fermentation was 99.84%. The relative abundance of Mucoromycota, Ascomycota, and Basidiomycota on the twelfth day of fermentation was different, at 94.89%, 3.8%, and 0.15%, respectively. The relative abundance of Mucoromycota, Ascomycota, and Basidiomycota on the eighteenth day of fermentation was different, at 83.78%, 12.20%, and 0. 37%, respectively. Among them, the relative abundance of Mucoromycota in fermented sufu on the third day was 99.99%. As can be seen from Figure 7, the two most dominant bacteria phyla in the fermentation process were Firmicutes and Proteobacteria, and their relative abundance was 99.61% and 0.29%, respectively, on the first day of fermentation. The proportions on the third day of fermentation were 33.16% and 1.12%, respectively. On the sixth day of fermentation, the proportions were 39.63% and 60.33%, respectively. On the twelfth day of fermentation, the proportions were 17.98% and 73.35%, respectively. On the eighteenth day of fermentation, the proportions were 16.09% and 67.24%, respectively. The proportion of Proteobacteria was 73.35% on the twelfth day of fermentation, which was the highest proportion. This decreased to 67.24% on the eighteenth day (*p* < 0.05). Firmicutes had the highest proportion—at 99.61%—on the third day, but this dropped to 39.63% on the sixth day.

**Figure 8:**
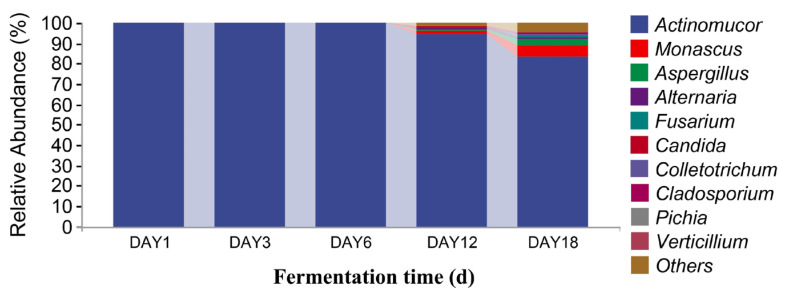
Distribution of fungal community structures in fermented Hongqu sufu samples at the genus level.

**Figure 9:**
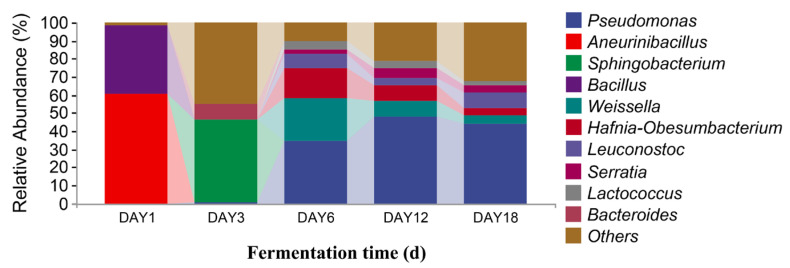
Distribution of bacterial community structures in fermented Hongqu sufu samples at the genus level.

In Figure 8, it can be seen that, at the genus level, there were 10 dominant fungi in sufu during fermentation: *Actinomucor*, *Monascus*, *Aspergillus*, *Alternaria*, *Fusarium*, *Candida*, *Colletotrichum*, *Cladosporium*, *Pichia*, and *Verticillium*. The relative abundance of *Actinomucor* on the first day of fermentation accounted was 99.88%. The proportion of *Actinomucor* on the third day of fermentation was 99.99%. The relative abundance of *Actinomucor* on the sixth day of fermentation was 99.71%. On the twelfth day of fermentation, the relative abundance of *Actinomucor*, *Monascus*, *Aspergillus*, *Alternaria*, *Fusarium*, *Candida*, *Colletotrichum*, *Cladosporium*, and *Pichia* was 94.85%, 1.39%, 0.67%, 0.43%, 0.19%, 0.68%, 0.12%, 0.08%, and 0.13%, respectively. On the eighteenth day of fermentation, the relative abundance of *Actinomucor*, *Monascus*, *Aspergillus*, *Alternaria*, *Fusarium*, *Candida*, *Colletotrichum*, *Cladosporium*, *Pichia*, *and Verticillium* was 83.74%, 5.35%, 2.65%, 1.17%, 0.93%, 0.17%, 0.49%, 0.35%, 0.23%, and 0.35%, respectively. The highest proportion of *Actinomucor* was 99.99%, which was observed on the third day of fermentation. *Monascus* and *Aspergillus* were added on the twelfth and eighteenth days of fermentation. From Figure 9, it can be seen that there were 10 dominant bacterial genera in the level classification of sufu bacteria during fermentation: *Pseudomonas*, *Aneurinibacillus*, *Sphingobacterium*, *Bacillus*, *Weissella*, *Hafnia-Obesumbacterium*, *Leuconostoc*, *Serratia*, *Lactococcus*, and *Bacteroides*. The relative abundance ratios of *Pseudomonas*, *Aneurinibacillus*, *Bacillus*, *Weissella*, and *Leuconostoc* on the first day of fermentation were 0.31%, 60.14%, 37.96%, 0.04%, 0.05%, and 0.06%, respectively. The relative abundance of *Pseudomonas*, *Sphingobacterium*, *Leuconostoc*, and *Bacteroides* on the third day of fermentation was 0.13%, 45.97%, 0.03%, and 8.98%, respectively. On the sixth day of fermentation, the relative abundance of *Pseudomonas*, *Weissella*, *Hafnia-Obesumbatch*, *Leuconostoc*, *Serratia*, and *Lactococcus* was 34.60%, 23.17%, 16.68%, 7.90%, 2.33%, and 4.88%, respectively. On the twelfth day of fermentation, the relative abundance of *Pseudomonas*, *Bacillus*, *Weissella*, *Hafnia-Obesumbatch*, *Leuconostoc*, *Serratia*, and *Lactococcus* was 47.52%, 0.01%, 9.15%, 8.72%, 3.99%, 5.13%, and 3.89%, respectively. On the eighteenth day of fermentation, the relative abundance of *Pseudomonas*, *Bacillus*, *and Weissella*, *Hafnia-Obesumbacterium*, and *Leuconostoc* was 44.16%, 0.02%, 4.53%, 4.14%, 8.52%, 4.07%, 1.91%, and 0.01%, respectively. The proportion of Pseudomonas in the fermented sufu on the twelfth day of fermentation was 47.52%, which was the highest proportion. On the eighteenth day of fermentation, the proportion decreased to 44.16% (*p* < 0.05). *Serratia* and *Bacteroides* were fermented on the twelfth and eighteenth days, respectively, when they were present. They were almost absent on fermentation days one, three, and six. On the twelfth and eighteenth days—the late fermentation period—*Serratia* accounted for 5.13% and 4.07%, respectively. A comprehensive analysis yielded *Pseudomonas*, *Serratia*, and *Bacteroides,* suggesting that the three make a major contribution to the special flavor of Hongqu sufu.

### 3.4. Analysis of Flavor Compounds in Sufu

HS-SPME-GC-MS was used to analyze the volatile flavor compounds at different stages of fermentation, and the mass spectra collected in the sufu samples through HS-SPME-GC-MS were retrieved using the mass spectrometry library [22,23,24]. The results are shown in Table 1.

As can be seen from Table 1, 75 volatile compounds were detected by HS-SPME-GC-MS in the sufu samples, including seven types of alcohol, four types of acid, 16 alkanes, 14 olefins, seven types of aldehydes, two ketones, 10 esters, one species of phenol, one sulfur-containing compound, one benzene, and 12 species of other compounds. At each stage of fermentation, five types of volatile compounds and two types of alcohol (aminoethanol and 3-methyl-1-butanol) were identified in the sufu. There were also two types of alkanes: cyclopentasiloxane and octamethylcyclotetrasiloxane. There was also one other compound: carboxymethylcellulose. Furthermore, in the late fermentation stage, diethylene glycol, 4-(1-methylethyl)-benzaldehyde, humulene, anethene, 3.7-base–1.6-octadiene, hexanoic acid, ethyl ester, cis-1,2,3,5,6,8-hexahydro-4, and 7-dimethyl-1-(methyl ether)nae were found to increase, as well as other flavor compounds. Among these, diethylene glycol has no peculiar smell and has a spicy sweetness. Humulene has a unique aroma, which is often present in the beer production process, meaning that beer presents a unique aroma. Anethene has an aroma of fennel, licorice, and camphor. Due to their special odors, hexanoic acid and ethyl ester are commonly used in apple, banana, and other fruity flavors. Chen Xinhua et al. [25] found that some flavor compounds in sufu disappeared in the late stages of fermentation. They also found that olefin content was significantly reduced and that new flavor compounds were also produced. These were analyzed, showing that these flavor compounds appeared in the late fermentation stage and that they provided a special odor for Hongqu sufu.

### 3.5. Correlation of the Flora Structure of Hongqu Sufu with Flavor Compounds

**Figure 10:**
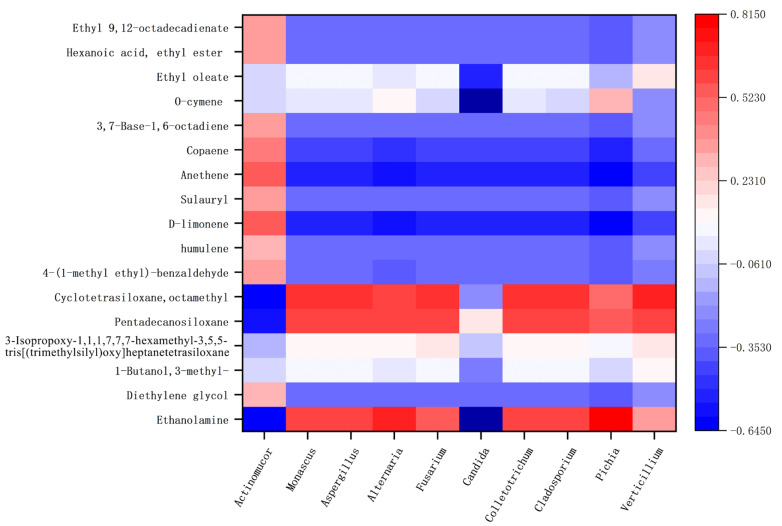
Thermogram of the relationship between the colony structure and characteristic flavor of fermented sufu and fungi.

**Figure 11:**
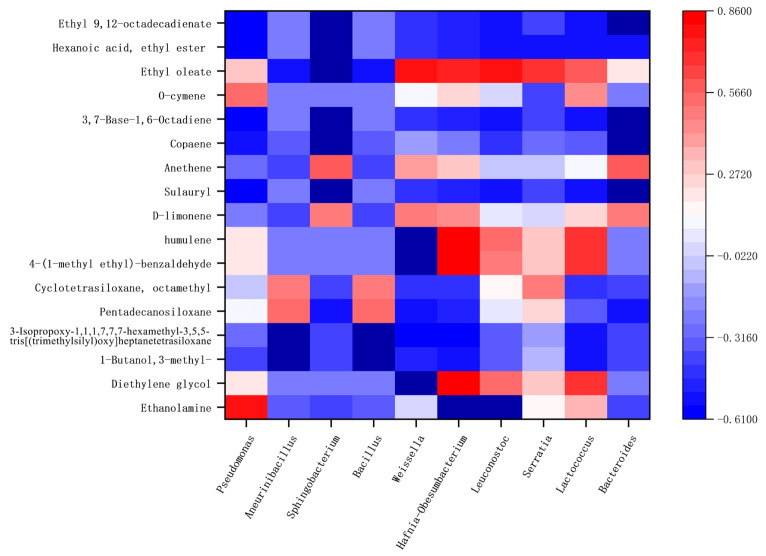
Thermogram of bacterial colony structures and the characteristic flavor of fermented sufu after fermentation.

Correlation between the flora in Hongqu sufu and special flavor compounds was used to make a heat map, as shown in Figure 10 and Figure 11. From this, positive and negative correlations between the structure of sufu’s flora and its flavor compounds could be obtained. In Figure 10 and Figure 11, red represents a positive correlation, blue represents a negative correlation, and white represents a correlation of zero. Figure 10 shows that the fungi genera *Alternaria* and *Pichia* were significantly related to the most volatile flavor components. *Candida* was mostly negatively correlated with esters, alcohols, and alkane content. As can be seen from Figure 11, the bacteria belonging to *Weissella*, *Hafnia-Obesumbacterium*, and *Leuconostoc* had a strong positive correlation with ethyl oleate. Most of these compounds provide winey, floral, and fruity aromas to sufu [26,27]. Most of them were spicy and slightly sweet, and they all provide an essential and unique smell and flavor to Hongqu sufu. However, two species of fungi, *Alternaria* and *Pichia*, and two species of bacteria, *Hafnia Obesumbacterium* and *Leuconostoc*, only appeared on the twelfth and eighteenth days, which was late in the fermentation process. This indicates that this type of bacteria is the main provider of the flavor compounds in Hongqu sufu. This is consistent with the speculations from previous articles.

## 4. Conclusions

Based on HTS and HS-SPME-GC-MS, the microbial flora structure and special flavor compounds of Hongqu sufu were comprehensively analyzed in this study. The results showed that, at the phylum level, there were fungal phyla (Mucoromycota, Ascomycota, Basidiomycota, Rozellomycota, Mortierellomycota, and Glomeromycota) and two bacterial phyla (Firmicutes and Proteobacteria). At the genus level, there were 10 species of fungi: *Actinomucor*, *Monascus*, *Aspergillus*, *Alternaria*, *Fusarium*, *Candida*, *Colletotrichum*, *Cladosporium*, *Pichia*, and *Verticillium*. There were also 10 species of bacteria: *Pseudomonas*, *Aneurinibacillus*, *Sphingobacterium*, *Bacillus*, *Weissiella*, *Hafnia-Obesumbacterium*, *Leuconostoc*, *Serratia*, *Lactococcus*, and *Bacteroides*. The similarities among the microorganisms in Hongqu sufu during fermentation were relatively small, but the differences were significant. The fungi genera *Alternaria* and *Pichia* were significantly associated with the most volatile flavor components. *Candida* was mostly negatively correlated with the flavor compounds. The bacteria genera *Weissella*, *Hafnia-Obesumbacterium,* and *Leuconostoc* had a strong positive correlation with ethyl oleate. The bacterial genus with the highest proportion in Hongqu sufu on the twelfth day of fermentation was *Pseudomonas*. The bacteria with the highest levels on the eighteenth day were *Weissella*, *Hafnia-Obesumbacterium*, and *Leuconostoc*. These were most correlated with the volatile flavor components and provide a special flavor for Hongqu sufu. In this study, changes in the diversity of microbial flora and the characteristic flavors of Hongqu sufu at different fermentation periods were analyzed. Through this, changes in the diversity of Hongqu sufu’s structure and the correlation with special flavor compounds were explored. This was helpful in analyzing the formation mechanism of the flavor compounds throughout the structure of Hongqu sufu, which could help to improve the flavor, quality, and stability of these products, providing a basis for traditional industries.

## Figures and Tables

**Table 1:** Relative content of the main flavor compounds in fermented Hongqu sufu.

Serial Number	Category	Compounds	Fermentation Time (d)/Relative Content (%)				
			1	3	6	12	18
1	Alcohols	Ethanolamine	0.47	3.41	2.31	11.65	7.49
2		Diethylene glycol	-	-	0.38	2.43	1.98
3		1-Hexanol	-	-	-	0.38	-
4		2,3-Butanediol	1.87	-	-	-	1.11
5		3-Hydroxy-butanol	1.27	2.32	1.20	0.08	0.13
6		3-Octanol	-	-	-	-	0.03
7		3-Methyl-1-butanol	14.63	-	-	1.18	5.66
8	Acids	Acetic acid	-	-	-	-	11.65
9		Pteroxate-6-carboxylic acid	-	-	-	0.06	-
10		2-Hydroxyethyl propionic acid	-	0.21	-	-	-
11		3-Aminobutyric acid	-	-	-	0.04	6.15
12	Alkanes	Methyl silane	0.68	-	-	0.49	-
13		Silane	0.32	-	-	1.41	0.46
14		3-Isopropoxy-1,1,1,7,7,7-hexamethyl-3,5,5-tris[(trimethylsilyl)oxy]heptanetetrasiloxane	-	0.76	0.65	1.07	1.38
15		Pentadecano siloxane	11.21	0.56	2.32	5.66	11.49
16		Cyclotetrasiloxane octamethyl	3.32	0.02	0.04	0.05	4.11
17		Decamethyltetra siloxane	-	0.56	-	0.18	0.21
18		Dodecamethyl cyclohexasiloxane	0.61	1.55	1.43	3.98	-
19		Dodecamethyl hexasiloxane	-	-	-	-	7.16
20		Dodecamethyl pentasiloxane	0.41	-	-	0.4	0.69
21		Tetradecane	0.32	-	-	0.20	0.16
22		Cetyltetramethyl hexasiloxane	0.55	0.25	-	0.52	-
23		Cetylhepta siloxane	0.25	-	-	0.26	-
24		Hexamethyl oxane	-	0.73	-	-	-
25		Heptadecane	0.10	-	-	-	-
26		2-Methyltetradecane	0.07	0.08	-	-	-
27		3-Trifluoroacetoxypentadecane	0.09	-	-	-	0.10
28	Aldehydes	Hyacinthin	0.31	-	-	-	-
29		Hexanal	0.24	-	-	1.31	1.49
30		Nonanaldehyde	-	-	-	0.16	0.26
31		2,4-Decadienal	-	-	-	0.04	0.6
32		2-Heptenal	0.31	-	-	-	0.12
33		3-Hydroxy-butyraldehyde	0.23	-	-	-	0.06
34		4-(1-methyl ethyl)-benzaldehyde	-	-	1.62	2.45	2.19
35	Ketones	L-Fenone	-	0.09	-	-	-
36		4-Methyl-1-(1-methylethyl)-3-cyclohexanone	-	0.06	-	-	-
37	Alkenes, hydrocarbons	Sesquicyclene	1.32	-	0.12	-	-
38		Humulene	-	-	0.08	-	-
39		D-Limonene	-	0.60	0.75	-	-
40		Cressene	-	-	1.76	1.09	2.93
41		Sulauryl	-	0.16	-	-	-
42		Anethene	-	-	0.02	0.12	1.21
43		Copaene	-	0.24	0.07	-	-
44		Nonadecatriene	0.08	-	-	-	0.06
45		1-2-Dimethyl-4-(1-methylethyl)-1,4-Cyclohexadiene	-	0.05	-	-	-
46		Vinyl-1-methyl-2,4-cyclohexane-1-methylbisethylene	-	0.08	-	-	-
47		2-Methyl-1-tetradecene	-	0.09	0.06	-	-
48		2-Octene	0.65	-	-	0.21	0.29
49		3.7-Base-1.6-Octadiene	-	-	1.21	1.12	0.11
50		o-Cymene	-	0.17	0.25	27.08	-
51	Esters	Ethyl Oleate	-	-	-	-	0.17
52		Hexanoic acid, ethyl ester	-	-	0.08	1.76	1.31
53		Ethyl octanoate	0.16	-	-	0.14	0.04
54		Ethyl tetradecanoate	-	-	0.09	-	-
55		Ethyl hexadecanoate	-	-	-	0.02	-
56		Ethyl octadecanoate	-	-	0.71	-	0.36
57		[1,1′-Dicyclopropyl]-2-octanoic acid 2′-hexyl methyl ester	0.23	-	-	0.05	0.02
58		Methyl 9,12-octadecenoate	-	-	-	0.70	0.39
59		Hexanoic acid, ethyl ester	-	10.70	-	-	-
60		Methyl 12,15-octadecenoate	0.15	18.38	-	0.03	0.03
61	Benzene	Pentylbenzene	0.18	-	-	-	-
62	Sulfur	Cyclic eight-membered sulfur	0.12	-	-	0.03	0.08
63	Phenols	Methylpiperol	-	0.91	-	-	-
64	Other	2-Pentyl-furan	6.14	-	-	4.52	7.33
65		2-Butylfuran	-	-	-	-	0.13
66		Alanylglycine dipeptide	3.79	-	-	4.24	6.13
67		cis-1,2,3,5,6,8-Hexahydro-4,7-dimethyl-1-(methyl ether)nae	-	-	1.75	3.69	2.76
68		2-Bromooctadecycarbon A Nayl	-	-	-	-	0.06
69		Oxymethoxyphenyl	-	-	-	-	0.10
70		Carboxymethylcellulose	1.23	0.21	0.20	0.17	0.24
71		Trimethylsiloxane borate	-	-	-	0.02	-
72		Bis(trimethylsilyl) Thioglycolates	0.14	-	-	0.16	0.57
73		9,12-Octadecacarbon dienyl chloride	1.24	-	-	1.02	0.39
74		Guanosine	-	-	-	-	0.03
75		Topotecan	0.87	-	-	0.30	-

Note: - means not detected.

## Data Availability

The datasets generated for this study are available on request to the corresponding author.
